# Associations of smartphone addiction and physical activity with sleep quality and neck/shoulder symptoms in university students: a cross-sectional study

**DOI:** 10.3389/fpubh.2026.1848640

**Published:** 2026-06-22

**Authors:** Wei Yue, Shen Cao, Jinglei Zhao, Qin Wang, Weigang Lai, Jinhui Zhang, Yuexin Cui

**Affiliations:** 1 College of Physical Education, Jilin Normal University, Siping, Jilin, China; 2 Football School, Hebei Institute of Communications, Shijiazhuang, Hebei, China; 3 School of Culture, Tourism, Health and Wellness Education, Dongying Vocational College, Dongying, Shandong, China; 4 Sports Committee, Dongying Vocational College, Dongying, China; 5 Faculty of Education and Liberal Studies, City University Malaysia, Kuala Lumpur, Malaysia; 6 Office of Academic Affairs, Dongying Vocational College, Dongying, China

**Keywords:** neck/shoulder symptoms, physical activity, sedentary behaviour, sleep quality, smartphone addiction

## Abstract

**Objective:**

To examine the associations of smartphone addiction, physical activity, and sedentary time with sleep quality and neck/shoulder symptoms during the previous 7 days in university students, and to assess the potential modifying role of physical activity in outcomes associated with smartphone addiction.

**Methods:**

A cross-sectional survey was conducted among 585 students from a single university in Jilin Province, China. Smartphone addiction, physical activity, sedentary time, sleep quality, and neck/shoulder symptoms were assessed using validated questionnaires. Multivariable linear regression and Poisson regression with robust variance were used to examine adjusted associations and interaction terms.

**Results:**

Of the participants, 43.8% were classified as having poor sleep, and 46.2% reported at least one neck/shoulder symptoms during the previous 7 days. After adjustment for covariates, higher smartphone addiction and longer sedentary time were both associated with higher PSQI scores, a higher prevalence of poor sleep, and a higher prevalence of neck/shoulder symptoms during the previous 7 days. Meeting physical activity recommendations was associated with lower PSQI scores and a lower prevalence of poor sleep, but showed no independent association with neck/shoulder symptoms. After further adjustment for PSQI, smartphone addiction, sedentary time, and PSQI score were still significantly associated with a higher prevalence of neck/shoulder symptoms. Interaction plots showed descriptively lower predicted PSQI scores and lower predicted probabilities of neck/shoulder symptoms among students who met physical activity recommendations at comparable levels of smartphone addiction; however, the formal interaction terms were not statistically significant.

**Conclusion:**

Smartphone addiction and sedentary time were consistently associated with poorer sleep quality and more frequent neck/shoulder symptoms. Physical activity was more consistently associated with sleep outcomes than with neck/shoulder symptoms. These findings support considering smartphone use, sedentary behaviour, physical activity, and sleep management together in campus health promotion research.

## 1 Introduction

University students may experience multiple health-related challenges, including sleep problems, prolonged sedentary behaviour, and musculoskeletal discomfort. Current epidemiological evidence indicates that the overall detection rate of sleep disturbance among Chinese university students has reached 25.7% (1), while the global prevalence of insomnia symptoms among undergraduates is close to 50% (2). At the same time, university students spend approximately 7.29 h/day sedentary on average representing a relatively high level of sedentary behaviour among young adults. Musculoskeletal problems also warrant attention. Data from the Global Burden of Disease (GBD) 2021 study indicate that approximately 203 million people worldwide were living with neck pain in 2020, and this number is projected to rise to 269 million by 2050 (3, 4). In university students, the risk factors for neck pain have become increasingly clear. Insufficient physical activity, prolonged use of electronic devices, sustained neck flexion, late sleeping, a history of neck or shoulder injury, female sex, and senior academic standing have all been associated with increased risk, with particularly strong associations observed for insufficient physical activity, prolonged head-down posture, and prior injury (5). In campus settings increasingly shaped by screen-based learning and sedentary lifestyles, impaired sleep and neck/shoulder musculoskeletal discomfort should be regarded as health problems of clear preventive relevance in university populations, rather than as isolated or occasional individual complaints.

In research on student health, smartphone addiction, particularly smartphone application-based addictive symptoms, has gradually emerged as a behavioural exposure of concern, although measurement tools and diagnostic thresholds remain inconsistent across studies (6). With respect to sleep outcomes, the epidemiological evidence is relatively consistent: excessive smartphone use is stably associated with poorer sleep quality, and longer daily use is linked to a higher risk of adverse sleep outcomes (7). This pattern has also been observed in university populations. A study of Chinese university students based on objective smartphone-use records found that students with smartphone addiction had a substantially higher risk of poor sleep and correspondingly shorter nocturnal sleep duration (8). At the same time, physical activity is not merely an independent factor outside this relationship. Studies in university samples suggest that physical activity may be related to the association between smartphone addiction and sleep quality, and that smartphone addiction may also be involved in the broader behavioural relationship between physical activity and sleep quality (9, 10). By comparison, the evidence linking smartphone use to musculoskeletal outcomes has accumulated more slowly. Nonetheless, available studies have shown that excessive smartphone use is associated with an increased risk of neck pain (11), and among university students it coexists with neck and upper-extremity pain and functional limitation (12). To date, most studies have focused separately on either sleep or neck pain, and evidence remains limited for the joint examination of physical activity, sedentary behaviour, sleep quality, and neck/shoulder symptoms within a single analytical framework.

Regarding neck/shoulder symptoms, the current evidence base is dominated by epidemiological findings on neck pain. Studies in adults suggest that sedentary exposure is associated with an increased risk of neck pain, and this association appears to be more pronounced in university students (13). Further evidence indicates a dose–response pattern, whereby longer sedentary time is associated with higher neck pain risk, with particularly evident associations for smartphone-related sedentary behaviour (14). In university students, insufficient physical activity, late sleeping, prolonged use of electronic devices, sustained neck flexion, and a history of neck or shoulder injury have all been associated with neck pain (5). Longitudinal follow-up studies further suggest that poorer sleep quality and insufficient physical activity predict subsequent neck pain (15). Together, these findings indicate that smartphone-related behaviours, physical activity, sedentary behaviour, sleep, and neck/shoulder symptoms are not isolated from one another, but are more likely embedded within the same lifestyle-related risk structure. However, most existing studies remain limited to single exposure-single outcome models or pairwise associations, and evidence remains scarce for simultaneously examining these factors within a unified analytical framework in university students.

Guided by a campus lifestyle-related health framework, smartphone addiction, sedentary behaviour, physical activity, sleep quality, and neck/shoulder symptoms were considered as interrelated but not causally ordered components of student health (Supplementary Figure S1). Smartphone addiction was considered relevant to sleep because it may co-occur with bedtime screen exposure and reduced sleep opportunity; sedentary behaviour was treated as distinct from insufficient physical activity because students may meet physical activity recommendations while still spending prolonged time sitting; and sleep quality and neck/shoulder symptoms were examined together because discomfort, fatigue, and recovery-related factors may co-occur in daily student life.

Based on this framework, the present cross-sectional study examined the associations of smartphone addiction (In this manuscript, the term “smartphone addiction” is used consistently to refer to smartphone application-based addictive symptoms measured by the SABAS, and should not be interpreted as a formal clinical diagnosis), physical activity, and sedentary behaviour with sleep quality and neck/shoulder symptoms, and tested whether the associations between smartphone addiction and these outcomes differed by physical activity status. We hypothesised that higher smartphone addiction and longer sedentary time would be associated with poorer sleep quality and more frequent neck/shoulder symptoms, whereas meeting physical activity recommendations would be associated with more favourable sleep-related outcomes. Given the cross-sectional design, this study was intended to identify association patterns rather than infer causal or temporal relationships.

## 2 Materials and methods

### 2.1 Study design and setting

This cross-sectional survey was conducted at a single university in Jilin Province, China, from December 2025 to February 2026. Data were collected using a standardised structured questionnaire administered on campus to assess smartphone addiction, physical activity, sedentary behaviour, sleep quality, and neck/shoulder symptoms among university students. The study was conducted at one university only, and no participants were recruited from other universities or provinces. Therefore, the sample should be regarded as an institution-based convenience sample rather than a representative sample of Chinese university students. The manuscript was prepared and reported in accordance with the basic requirements of the Strengthening the Reporting of Observational Studies in Epidemiology (STROBE) statement (16).

### 2.2 Participants and sampling

Eligible participants were undergraduate and postgraduate students enrolled at the study university during the survey period who were able to understand and complete the questionnaire independently, reported regular smartphone use on most days during the previous month, and provided complete data on the main study variables. To minimise the influence of overt physical conditions on the assessment of musculoskeletal symptoms and sleep, students were excluded if they had experienced an acute neck/shoulder injury or undergone related surgery recently, or if they self-reported severe neurological disease, rheumatic or autoimmune disease, or other serious physical illness that might substantially affect musculoskeletal symptoms or sleep status.

Participants were recruited using non-probability convenience cluster sampling at the class level. Classes were selected according to fieldwork accessibility rather than random sampling, and no sampling weights were applied. All students present in the selected classes were invited to participate voluntarily. A total of 630 students were approached, of whom 597 returned questionnaires, yielding a questionnaire return rate of 94.8%. After excluding 12 questionnaires with missing data on key variables, 585 students were included in the final analysis, corresponding to a valid questionnaire rate of 92.9% among approached students and 98.0% among returned questionnaires.

A complete-case analysis strategy was used because only 12 returned questionnaires were excluded for missing key variables. Multiple imputation was not applied because the proportion of missing data was small and the missingness involved variables required for the prespecified primary models. Because this was a convenience-sampled cross-sectional survey, no formal *a priori* sample size calculation was conducted. The sample size was determined pragmatically by the classes accessible during the survey period and by the number of students available during on-site recruitment; it was not intended as a census of all eligible students at the university. The final sample size was considered adequate for the planned multivariable models, given the number of participants with poor sleep and neck/shoulder symptoms and the number of covariates included in the models.

### 2.3 Data collection procedures

Data were collected using paper-based questionnaires administered during uniformly organised class sessions or designated group-completion periods. Before distributing the questionnaires, researchers explained the study purpose, completion requirements, principles of anonymity, and the voluntary nature of participation. Participants proceeded to the formal questionnaire only after reading the informed consent information and confirming their agreement to participate. Questionnaires were completed independently by the students and collected on site. No directly identifiable personal information, such as names, student numbers, or contact details, was collected.

### 2.4 Measures

#### 2.4.1 Smartphone addiction, physical activity, and sedentary behaviour

Smartphone addiction was assessed using the Chinese version of the Smartphone Application-Based Addiction Scale (SABAS-CV) (17). The scale comprises six items rated on a 6-point Likert scale, with each item scored from 1 to 6, yielding a total score ranging from 6 to 36. Higher scores indicate more pronounced smartphone application-related addictive symptoms. To complement this measure with information on daily smartphone-use behaviour, the questionnaire also recorded average smartphone use on weekdays and weekends, expressed as hours/day. In the present study, the SABAS-CV showed good internal consistency, with a Cronbach’s *α* of 0.876.

Physical activity and sedentary behaviour were assessed using the Global Physical Activity Questionnaire (GPAQ). Following the standard GPAQ analytic protocol, moderate- to vigorous-intensity physical activity across work, transport, and leisure domains was summed. Moderate-intensity activity was assigned 4.0 metabolic equivalents (METs) and vigorous-intensity activity 8.0 METs to calculate total physical activity volume (MET-min/week). In accordance with the World Health Organization recommendations for adult physical activity, participants accumulating at least 600 MET-min/week were classified as meeting physical activity recommendations (meet_pa_guideline), whereas those below this threshold were classified as not meeting the recommendations. Sedentary behaviour was assessed using the GPAQ item on time spent sitting or reclining on a typical day, converted to hours/day, and analysed as a continuous variable (18, 19).

#### 2.4.2 Sleep quality and neck/shoulder symptoms

Sleep quality was assessed using the PSQI. We used PSQI > 7 as the primary cut-off because this threshold has been commonly used in Chinese populations and provides a conservative definition of poor sleep. To address the potential influence of cut-off selection, PSQI > 5 was further examined in sensitivity analyses. This instrument evaluates sleep over the previous month across subjective sleep quality, sleep latency, sleep duration, sleep efficiency, sleep disturbances, use of sleep medication, and daytime dysfunction, with total scores ranging from 0 to 21. Higher scores indicate poorer sleep quality (20, 21). In this study, the PSQI total score (psqi_total) was analysed as a continuous outcome. In addition, based on the commonly used Chinese cut-off, a PSQI total score > 7 was defined as poor sleep for binary analyses, consistent with common practice in Chinese students.

Neck/shoulder symptoms were assessed using relevant items from the Nordic Musculoskeletal Questionnaire (NMQ) (22, 23). Because the NMQ assesses the neck and shoulders as separate anatomical regions, neck/shoulder symptoms were first extracted separately. For the primary analysis, these two regions were combined into a composite neck/shoulder symptoms variable because they are anatomically adjacent upper-body regions and symptoms in these areas may co-occur in screen-based study and smartphone-use contexts. Participants reporting pain, soreness, discomfort, numbness, or related symptoms in either the neck or shoulder during the previous 7 days were classified as having any neck/shoulder symptoms in the previous 7 days. To describe the anatomical distribution of musculoskeletal discomfort, symptom information for different body regions over the previous 7 days and previous 12 months was also compiled for supplementary analyses of symptom reporting by body site.

#### 2.4.3 Covariates

Covariates were selected *a priori* before statistical analysis on the basis of previous literature, study hypotheses, and potential confounding factors, and were included to control for the possible influence of demographic characteristics, recent health behaviours, and underlying health status on the primary associations, rather than being introduced *post hoc* according to model results. Covariates included sex, age, academic year, and body mass index (BMI), the latter calculated from self-reported weight (kg) divided by height squared (m^2^). Age and BMI were treated as continuous variables, sex as a binary variable, and academic year was categorised as first year, second year, third year, fourth year, and postgraduate or above.

Smoking and alcohol consumption during the previous 30 days were also included to reflect recent health behaviours; both were classified on the basis of self-report as never, occasional, or frequent. Chronic disease status and history of neck/shoulder injury were additionally included as health-related covariates and were both coded as yes/no variables. All covariates were self-reported and entered into the regression models according to a prespecified coding scheme.

### 2.5 Statistical analysis

Sample characteristics were first described. Continuous variables were presented as mean ± standard deviation or median (interquartile range), depending on their distribution, and categorical variables as counts and percentages. Group comparisons were stratified by poor sleep and by any neck/shoulder symptoms during the previous 7 days. Normally distributed continuous variables were compared using independent-samples t-tests, skewed variables using the Wilcoxon rank-sum test, and categorical variables using the Pearson χ^2^ test; Fisher’s exact test was used when expected cell counts were too small to satisfy the assumptions of the χ^2^ test. Internal consistency of the SABAS-CV was evaluated by calculating Cronbach’s *α* across the six SABAS items among participants included in the final analytic sample.

To examine the independent associations between the main exposures and outcomes, three multivariable regression models were constructed. Model 1 used multivariable linear regression with PSQI total score as the dependent variable. Model 2 used Poisson regression with robust variance with poor sleep as the dependent variable. Model 3 also used Poisson regression with robust variance, with any neck/shoulder symptoms during the previous 7 days as the dependent variable. Because the binary outcomes were not rare in this study, Poisson models with robust variance were used to estimate prevalence ratios (PRs) and their 95% confidence intervals (CIs) directly. All multivariable models included the prespecified covariates of sex, age, academic year, BMI, smoking during the previous 30 days, alcohol consumption during the previous 30 days, chronic disease, and history of neck/shoulder injury. Model 3 further included PSQI total score to assess the associations of smartphone addiction, physical activity, and sedentary behaviour with neck/shoulder symptoms independently of sleep quality. Linear regression results are reported as *β* coefficients with 95% CIs, and Poisson regression results as PRs with 95% CIs (23, 24).

For interaction analyses, a product term between SABAS total score and meeting physical activity recommendations was added to the relevant regression models to test whether the associations of smartphone addiction with sleep quality or neck/shoulder symptoms differed by physical activity status. The coefficient, 95% CI, and *p* value for the interaction term were reported. For the robust Poisson model, the exponentiated interaction coefficient was reported as the PR for interaction. These interaction analyses were interpreted as tests of statistical effect modification only, rather than as evidence of causal moderation or buffering. Interaction plots of predicted PSQI total scores and predicted probabilities of neck/shoulder symptoms were then generated on the basis of model estimates. Two sensitivity analyses were conducted using the same modelling strategies and covariate adjustment schemes as the primary analyses. First, poor sleep was redefined as PSQI > 5 instead of PSQI > 7. Second, the musculoskeletal outcome was redefined as any neck/shoulder symptoms during the previous 12 months instead of the previous 7 days. To address the potential loss of information from dichotomising physical activity, additional analyses were conducted using alternative physical activity specifications. Total physical activity volume was analysed as a continuous variable per 1,000 MET-min/week, as quartiles, and with an exploratory quadratic term. Data management, statistical analyses, and figure generation were performed in R version 4.4.1. Benjamini–Hochberg FDR correction was applied to the 12 prespecified primary tests, including the main exposure–outcome associations and the two SABAS total score × physical activity interaction terms. Poisson model stability was assessed using model convergence, outcome events per estimated coefficient, sparse-cell checks, and collapsed smoking/alcohol sensitivity analyses.

### 2.6 Timeframe differences between instruments

In this study, sleep quality was assessed using the PSQI, which evaluates sleep over the previous 30 days, whereas musculoskeletal symptoms were assessed using the NMQ, which evaluates symptoms over the previous 7 days. We chose these validated instruments with their original recall periods to maintain psychometric reliability and comparability with prior studies.

### 2.7 Ethics statement

This study involved human participants and was approved by the Medical Ethics Committee of the Second Hospital of Jilin University [Approval No. (2025) 412]. All participants provided informed consent before participation. Participation was voluntary, and participants were informed that they could withdraw at any time. The survey was conducted anonymously using paper-based questionnaires, and no directly identifiable personal information, such as names, student numbers, telephone numbers, or contact details, was collected. All data were used only for scientific research and were treated confidentially.

## 3 Results

### 3.1 Participant characteristics

Of the 597 returned questionnaires, 12 were excluded because of missing key variables; therefore, all analyses were based on 585 complete cases (Table 1). The mean age of the sample was 20.46 ± 1.66 years, 57.4% were female, and the mean body mass index (BMI) was 22.16 ± 3.37 kg/m^2^. Mean smartphone use was 4.52 ± 1.68 h/day on weekdays and 5.96 ± 2.24 h/day on weekends. The mean Smartphone Application-Based Addiction Scale (SABAS) total score was 21.22 ± 7.23. The median total physical activity volume assessed by the Global Physical Activity Questionnaire (GPAQ) was 1200.0 (560.0, 2380.0) MET-min/week, corresponding to a median total physical activity time of 258.0 (120.0, 476.0) min/week. Mean sedentary time was 7.51 ± 1.99 h/day. The mean Pittsburgh Sleep Quality Index (PSQI) total score was 7.36 ± 3.15, 43.8% of participants were classified as having poor sleep, and 46.2% reported at least one neck/shoulder symptoms during the previous 7 days. Overall, smartphone use and sedentary exposure were common in this sample, and sleep problems and neck/shoulder discomfort were also highly prevalent, providing a basis for the subsequent association analyses.

**Table 1:** Characteristics of the study participants.

Variable	Result
Age, years	20.46 ± 1.66
Height, cm	165.80 ± 7.61
Weight, kg	61.28 ± 12.33
Body mass index, kg/m^2^	22.16 ± 3.37
Smartphone use on weekdays, h/day	4.52 ± 1.68
Smartphone use on weekends, h/day	5.96 ± 2.24
Smartphone Application-Based Addiction Scale total score	21.22 ± 7.23
Global Physical Activity Questionnaire total physical activity, MET-min/week	1200.0 (560.0, 2380.0)
Total physical activity time, min/week	258.0 (120.0, 476.0)
Sedentary time, h/day	7.51 ± 1.99
Pittsburgh Sleep Quality Index total score	7.36 ± 3.15

Data are presented as mean ± standard deviation or median (interquartile range).

### 3.2 Comparison by sleep quality

When stratified by sleep quality, the poor-sleep group showed a less favourable behavioural and health profile (Table 2). Compared with the better-sleep group, students with poor sleep had higher levels of smartphone addiction, longer smartphone use on both weekdays and weekends, lower overall physical activity, and a markedly lower prevalence of meeting physical activity recommendations (60.9% vs. 83.6%). They also had significantly longer sedentary time and a substantially higher prevalence of any neck/shoulder symptoms during the previous 7 days (61.7% vs. 34.0%). The proportion of female was also higher in the poor-sleep group. By contrast, age, BMI, academic year, smoking, alcohol consumption, chronic disease, and history of neck/shoulder injury did not differ significantly between the two groups.

**Table 2:** Comparison of characteristics by sleep quality.

Variable	Overall (*N* = 585)	Good sleep (*N* = 329)	Poor sleep (*N* = 256)	*p* value
Age, years	20.46 ± 1.66	20.43 ± 1.67	20.50 ± 1.64	0.618
Body mass index, kg/m^2^	22.16 ± 3.37	22.18 ± 3.23	22.14 ± 3.54	0.886
Smartphone Application-Based Addiction Scale total score	21.22 ± 7.23	18.14 ± 6.24	25.17 ± 6.47	<0.001
Global Physical Activity Questionnaire total physical activity, MET-min/week	1200.0 (560.0, 2380.0)	1592.0 (832.0, 2924.0)	888.0 (295.0, 1648.0)	<0.001
Sedentary time, h/day	7.51 ± 1.99	6.72 ± 1.74	8.52 ± 1.83	<0.001
Smartphone use on weekdays, h/day	4.52 ± 1.68	4.04 ± 1.65	5.13 ± 1.53	<0.001
Smartphone use on weekends, h/day	5.96 ± 2.24	5.34 ± 2.12	6.75 ± 2.13	<0.001
Female, *n* (%)	336 (57.4%)	172 (52.3%)	164 (64.1%)	0.004
Meeting physical activity recommendations, *n* (%)	431 (73.7%)	275 (83.6%)	156 (60.9%)	<0.001
Any neck/shoulder symptom (past 7 days), *n* (%)	270 (46.2%)	112 (34.0%)	158 (61.7%)	<0.001

Data are presented as mean ± standard deviation, median (interquartile range), or number (percentage).

### 3.3 Comparison by neck/shoulder symptoms

When stratified by the presence of any neck/shoulder symptoms during the previous 7 days, the symptomatic group likewise exhibited a less favourable behavioural and health profile (Table 3). Compared with the asymptomatic group, these students had higher SABAS total scores, longer smartphone use on both weekdays and weekends, longer sedentary time, lower GPAQ total physical activity volume, and a lower prevalence of meeting physical activity recommendations (67.8% vs. 78.7%). They also had higher PSQI total scores, a markedly greater prevalence of poor sleep (58.5% vs. 31.1%), and a higher proportion of female. Age, BMI, academic year, history of neck/shoulder injury, smoking, alcohol consumption, and chronic disease did not differ significantly between the two groups.

**Table 3:** Comparison of characteristics by neck/shoulder symptoms (past 7 days).

Variable	Overall (*N* = 585)	No symptoms (*N* = 315)	Any symptoms (*N* = 270)	*p* value
Age, years	20.46 ± 1.66	20.41 ± 1.61	20.53 ± 1.72	0.38
Body mass index, kg/m^2^	22.16 ± 3.37	22.33 ± 3.22	21.96 ± 3.53	0.184
Smartphone Application-Based Addiction Scale total score	21.22 ± 7.23	19.06 ± 6.59	23.73 ± 7.15	<0.001
Global Physical Activity Questionnaire total physical activity, MET-min/week	1200.0 (560.0, 2380.0)	1416.0 (672.0, 2884.0)	1004.0 (442.0, 1787.0)	<0.001
Sedentary time, h/day	7.51 ± 1.99	6.88 ± 1.83	8.24 ± 1.92	<0.001
Smartphone use on weekdays, h/day	4.52 ± 1.68	4.15 ± 1.56	4.94 ± 1.72	<0.001
Smartphone use on weekends, h/day	5.96 ± 2.24	5.46 ± 2.06	6.54 ± 2.30	<0.001
Pittsburgh Sleep Quality Index total score	7.36 ± 3.15	6.40 ± 2.85	8.49 ± 3.12	<0.001
Female, *n* (%)	336 (57.4%)	167 (53.0%)	169 (62.6%)	0.02
Meeting physical activity recommendations, *n* (%)	431 (73.7%)	248 (78.7%)	183 (67.8%)	0.003
Poor sleep (PSQI > 7), *n* (%)	256 (43.8%)	98 (31.1%)	158 (58.5%)	<0.001

### 3.4 Multivariable linear regression for PSQI total score

After adjustment for sex, age, academic year, BMI, smoking, alcohol consumption, chronic disease, and history of neck/shoulder injury, multivariable linear regression showed that higher smartphone addiction and longer sedentary time were independently associated with higher PSQI total scores, whereas meeting physical activity recommendations was associated with lower PSQI total scores (Table 4). Specifically, each 1-point increase in SABAS total score was associated with a 0.163-point increase in PSQI total score (95% confidence interval [CI] 0.129 to 0.198; *p* < 0.001); each additional 1 h/day of sedentary time was associated with a 0.427-point increase in PSQI total score (95% CI 0.301 to 0.553; *p* < 0.001); and students who met physical activity recommendations had PSQI total scores that were, on average, 1.344 points lower than those who did not (95% CI -1.805 to −0.882; *p* < 0.001). In addition, female had higher PSQI total scores than male (*β* = 0.557, 95% CI 0.131 to 0.983; *p* = 0.010). Model fit was good, with an R^2^ of 0.437 and an adjusted R^2^ of 0.421. No statistically significant associations were observed for the remaining covariates.

**Table 4:** Multivariable linear regression analysis for Pittsburgh Sleep Quality Index total score.

Variable	β (95% CI)	*p* value
Age, years	−0.113 (−0.435, 0.210)	0.492
Body mass index, kg/m^2^	0.047 (−0.015, 0.110)	0.137
Chronic disease (yes vs. no)	0.150 (−0.822, 1.123)	0.761
Alcohol use (occasional vs. none)	−0.311 (−0.942, 0.320)	0.333
Alcohol use (frequent vs. none)	−0.223 (−0.899, 0.453)	0.517
Academic year (2 vs. 1)	−0.021 (−0.670, 0.628)	0.95
Academic year (3 vs. 1)	−0.011 (−0.843, 0.821)	0.979
Academic year (4 vs. 1)	0.073 (−0.999, 1.145)	0.894
Academic year (≥5 vs. 1)	0.294 (−1.415, 2.002)	0.736
Meeting physical activity recommendations (yes vs. no)	−1.344 (−1.805, −0.882)	<0.001
History of neck/shoulder injury (yes vs. no)	−0.191 (−1.022, 0.639)	0.651
Smartphone Application-Based Addiction Scale total score	0.163 (0.129, 0.198)	<0.001
Sedentary time (per hour/day)	0.427 (0.301, 0.553)	<0.001
Female (vs male)	0.557 (0.131, 0.983)	0.01
Smoking (occasional vs. none)	−0.630 (−1.467, 0.208)	0.14
Smoking (frequent vs. none)	−0.657 (−1.599, 0.285)	0.171

### 3.5 Robust Poisson regression for poor sleep

After adjustment for sex, age, academic year, BMI, smoking, alcohol consumption, chronic disease, and history of neck/shoulder injury, Poisson regression with robust variance showed that higher smartphone addiction and longer sedentary time were associated with a higher prevalence of poor sleep, whereas meeting physical activity recommendations was associated with a lower prevalence of poor sleep (Table 5). Specifically, each 1-point increase in SABAS total score was associated with a 5.5% higher prevalence of poor sleep (prevalence ratio [PR] 1.055, 95% CI 1.039 to 1.071; *p* < 0.001); each additional 1 h/day of sedentary time was associated with a 13.0% higher prevalence of poor sleep (PR 1.130, 95% CI 1.074 to 1.189; *p* < 0.001); and students who met physical activity recommendations had a 25.8% lower prevalence of poor sleep than those who did not (PR 0.742, 95% CI 0.628 to 0.877; *p* < 0.001). In addition, female had a higher prevalence of poor sleep than male (PR 1.242, 95% CI 1.032 to 1.496; *p* = 0.022). No statistically significant associations were found for the remaining covariates.

**Table 5:** Poisson regression with robust variance for poor sleep (PSQI > 7).

Variable	PR (95% CI)	*p* value
Smartphone Application-Based Addiction Scale total score (per 1-point increase)	1.055 (1.039, 1.071)	<0.001
Meeting physical activity recommendations (yes vs. no)	0.742 (0.628, 0.877)	<0.001
Sedentary time (per hour/day)	1.130 (1.074, 1.189)	<0.001
Female (vs male)	1.242 (1.032, 1.496)	0.022
Age, years	0.962 (0.845, 1.095)	0.556
Academic year (2 vs. 1)	0.987 (0.738, 1.322)	0.933
Academic year (3 vs. 1)	1.038 (0.738, 1.461)	0.83
Academic year (4 vs. 1)	1.077 (0.690, 1.681)	0.743
Academic year (≥5 vs. 1)	1.115 (0.564, 2.204)	0.754
Body mass index, kg/m^2^	1.020 (0.994, 1.047)	0.128
Smoking (occasional vs. none)	0.883 (0.624, 1.249)	0.482
Smoking (frequent vs. none)	0.995 (0.609, 1.625)	0.984
Alcohol use (occasional vs. none)	1.037 (0.814, 1.321)	0.77
Alcohol use (frequent vs. none)	0.848 (0.645, 1.114)	0.236
Chronic disease (yes vs. no)	1.099 (0.810, 1.490)	0.546
History of neck/shoulder injury (yes vs. no)	0.940 (0.620, 1.425)	0.771

*N* = 585.

### 3.6 Robust Poisson regression for neck/shoulder symptoms

After further inclusion of PSQI total score in the model, while also adjusting for sex, age, academic year, BMI, smoking, alcohol consumption, chronic disease, and history of neck/shoulder injury, SABAS total score, sedentary time, and PSQI total score remained independently associated with a higher prevalence of neck/shoulder symptoms during the previous 7 days (Table 6). Specifically, each 1-point increase in SABAS total score was associated with a 1.9% higher prevalence of neck/shoulder symptoms (PR 1.019, 95% CI 1.003 to 1.034; *p* = 0.019); each additional 1 h/day of sedentary time was associated with a 10.3% higher prevalence (PR 1.103, 95% CI 1.047 to 1.161; *p* < 0.001); and each 1-point increase in PSQI total score was associated with a 4.9% higher prevalence (PR 1.049, 95% CI 1.016 to 1.083; *p* = 0.003). By contrast, meeting physical activity recommendations was not significantly associated with neck/shoulder symptoms (PR 0.970, 95% CI 0.811 to 1.159; *p* = 0.736). None of the remaining covariates reached statistical significance.

**Table 6:** Poisson regression with robust variance for neck/shoulder symptoms (past 7 days).

Variable	PR (95% CI)	*p* value
Smartphone Application-Based Addiction Scale total score (per 1-point increase)	1.019 (1.003, 1.034)	0.019
Meeting physical activity recommendations (yes vs. no)	0.970 (0.811, 1.159)	0.736
Sedentary time (per hour/day)	1.103 (1.047, 1.161)	<0.001
Pittsburgh Sleep Quality Index total score (per 1-point increase)	1.049 (1.016, 1.083)	0.003
Female (vs male)	1.142 (0.953, 1.369)	0.149
Age, years	1.005 (0.890, 1.135)	0.934
Academic year (2 vs. 1)	0.894 (0.674, 1.184)	0.434
Academic year (3 vs. 1)	0.890 (0.644, 1.229)	0.478
Academic year (4 vs. 1)	0.858 (0.567, 1.298)	0.468
Academic year (≥5 vs. 1)	0.990 (0.523, 1.872)	0.974
Body mass index, kg/m^2^	0.992 (0.966, 1.019)	0.544
Smoking (occasional vs. none)	1.051 (0.739, 1.495)	0.781
Smoking (frequent vs. none)	0.983 (0.634, 1.524)	0.939
Alcohol use (occasional vs. none)	1.065 (0.825, 1.374)	0.629
Alcohol use (frequent vs. none)	0.731 (0.515, 1.039)	0.081
Chronic disease (yes vs. no)	0.955 (0.668, 1.366)	0.802
History of neck/shoulder injury (yes vs. no)	1.328 (0.983, 1.795)	0.064

*N* = 585.

### 3.7 Prevalence of musculoskeletal symptoms by body site

**Figure 1:**
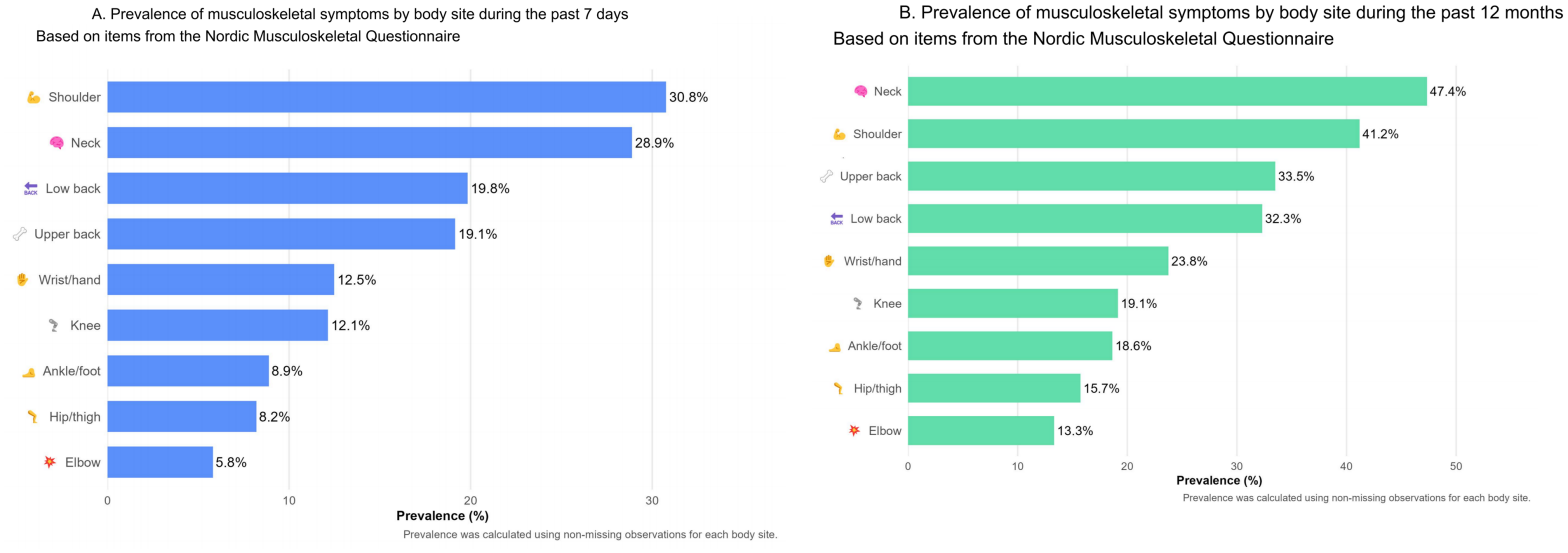
Prevalence of musculoskeletal symptoms by body site during the past 7 days and the past 12 months. Prevalence was calculated using non-missing observations for each body site based on the Nordic Musculoskeletal Questionnaire. Panel **(A)** shows symptom prevalence during the past 7 days, and panel **(B)** shows symptom prevalence during the past 12 months.

As shown in Figures 1A,B, musculoskeletal symptoms were concentrated primarily in the neck, shoulder, and back regions. During the previous 7 days, the shoulder and neck were the most commonly affected sites, with prevalences of 30.8 and 28.9%, respectively, followed by the lower back and upper back. Over the previous 12 months, this pattern was even more pronounced, with the prevalence rising to 47.4% for the neck and 41.2% for the shoulders; these remained the most frequently affected sites, followed by the upper back and lower back. Compared with the elbow, hip/thigh, and ankle/foot, symptoms were reported more frequently in the neck, shoulders, and back, indicating that musculoskeletal discomfort in this population was concentrated mainly in the upper body, particularly the neck/shoulder/back region.

### 3.8 Forest plots of adjusted associations

**Figure 2:**
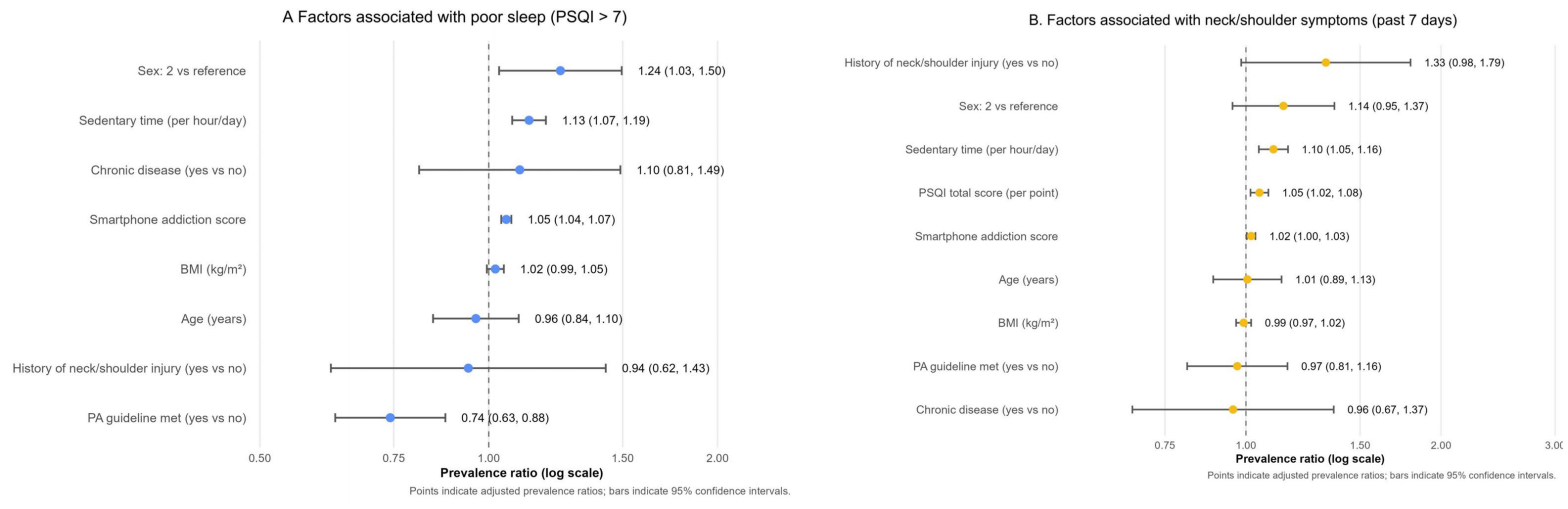
Forest plots of adjusted associations of smartphone addiction, physical activity, and sedentary time with poor sleep and neck/shoulder symptoms. Panel **(A)** shows adjusted prevalence ratios for poor sleep, defined as PSQI total score > 7. Panel **(B)** shows adjusted prevalence ratios for any neck/shoulder symptoms during the past 7 days. Models were adjusted for sex, age, academic year, body mass index, smoking during the previous 30 days, alcohol consumption during the previous 30 days, chronic disease, and history of neck/shoulder injury. The model for neck/shoulder symptoms was additionally adjusted for PSQI total score. Points indicate adjusted prevalence ratios, and horizontal bars indicate 95% confidence intervals.

Figures 2A,B provide a visual summary of the adjusted associations, with overall effect directions consistent with the regression results. Figure 2A shows that, after controlling for relevant confounders, higher smartphone addiction scores and longer sedentary time were positively associated with poor sleep, whereas meeting physical activity recommendations was negatively associated with poor sleep. Female also appeared more likely to have poor sleep. Figure 2B shows that factors associated with neck/shoulder symptoms were concentrated mainly in behavioural exposures and sleep status. Longer sedentary time, higher PSQI total score, and higher smartphone addiction score were all associated with a higher prevalence of neck/shoulder symptoms, with sedentary time and sleep showing relatively more stable associations. Across both forest plots, the point estimates for the main exposures were directionally consistent and the confidence intervals were relatively narrow, suggesting good stability of the model estimates.

### 3.9 Interaction between smartphone addiction and physical activity

The formal interaction analyses are presented in Supplementary Table S2. In the linear regression model for PSQI total score, the interaction term between SABAS total score and meeting physical activity recommendations was not statistically significant (*β* = −0.004, 95% CI -0.068 to 0.060; *p* = 0.895). Similarly, in the robust Poisson regression model for neck/shoulder symptoms during the previous 7 days, the interaction term was not statistically significant (PR for interaction = 1.007, 95% CI 0.984 to 1.031; *p* = 0.536).

**Figure 3:**
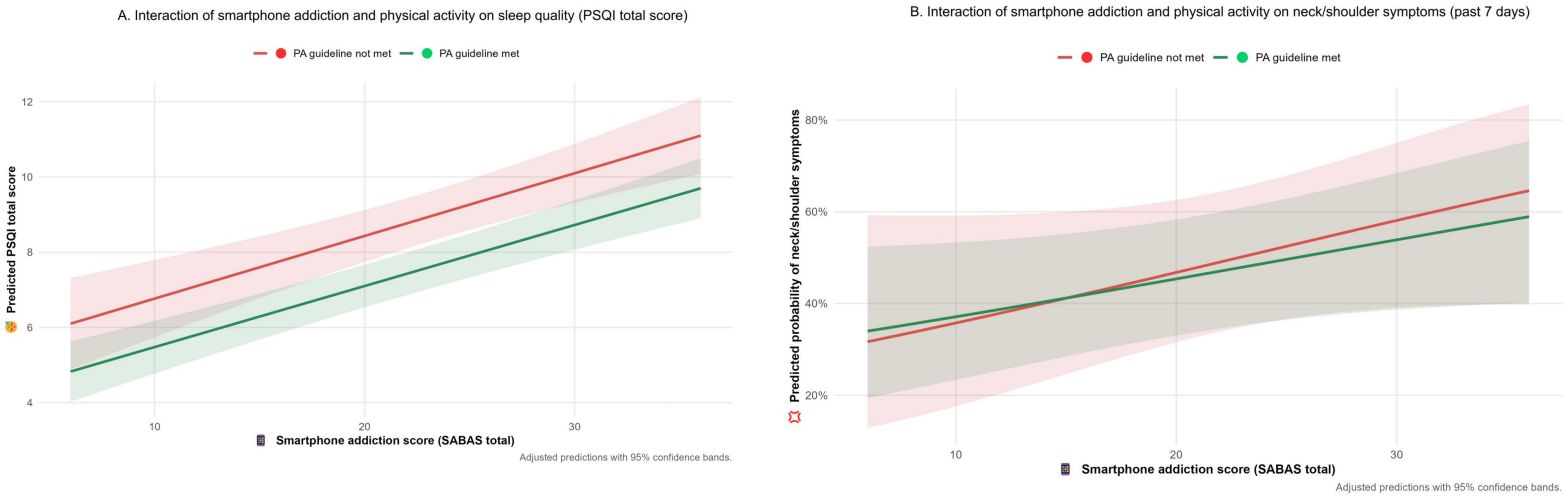
Interaction plots showing the joint associations of smartphone addiction and physical activity with predicted Pittsburgh Sleep Quality Index total score and predicted probability of neck/shoulder symptoms. Panel **(A)** shows predicted PSQI total scores from a linear regression model including the interaction term between Smartphone Application-Based Addiction Scale (SABAS) total score and meeting physical activity recommendations. Panel **(B)** shows predicted probabilities of neck/shoulder symptoms during the past 7 days from a robust Poisson regression model including the same interaction term. The PSQI model was adjusted for sedentary time, sex, age, academic year, body mass index, smoking during the previous 30 days, alcohol consumption during the previous 30 days, chronic disease, and history of neck/shoulder injury. The neck/shoulder symptoms model was additionally adjusted for PSQI total score. Shaded areas indicate 95% confidence bands.

As shown in Figure 3, predicted PSQI total scores and predicted probabilities of neck/shoulder symptoms were descriptively lower among students who met physical activity recommendations at comparable SABAS levels. However, because the formal interaction terms were not statistically significant, these graphical patterns should be interpreted descriptively rather than as evidence that physical activity buffered the associations of smartphone addiction with sleep quality or neck/shoulder symptoms. After FDR correction, the main conclusions were unchanged (Supplementary Table S4). The Poisson models converged successfully, showed adequate outcome events per coefficient, no major sparse-cell concern, and materially similar estimates after collapsing smoking and alcohol use.

### 3.10 Additional analyses of physical activity specification

Additional analyses using alternative physical activity specifications are shown in Supplementary Table S3. When total physical activity volume was analysed as a continuous variable, each additional 1,000 MET-min/week was associated with a lower PSQI total score (*β* = −0.261, 95% CI -0.393 to −0.129; *p* < 0.001) and a lower prevalence of poor sleep (PR 0.909, 95% CI 0.846 to 0.977; *p* = 0.010), but was not significantly associated with neck/shoulder symptoms during the previous 7 days (PR 0.960, 95% CI 0.900 to 1.024; *p* = 0.215). Analyses using physical activity quartiles showed similar patterns: higher quartiles were associated with lower PSQI total scores and, for Q3 and Q4, a lower prevalence of poor sleep, whereas no significant association was observed for neck/shoulder symptoms. Exploratory quadratic models suggested possible non-linearity for PSQI total score only, but not for poor sleep or neck/shoulder symptoms. Overall, these analyses supported the primary finding that physical activity was more consistently associated with sleep outcomes than with neck/shoulder symptoms.

### 3.11 Sensitivity analyses

Sensitivity analyses using alternative outcome definitions are presented in Supplementary Table S1. When poor sleep was redefined as PSQI > 5, SABAS total score, sedentary time, and meeting physical activity recommendations remained significantly associated with poor sleep, consistent with the primary analysis. When neck/shoulder symptoms were redefined as symptoms during the previous 12 months, SABAS total score and sedentary time remained significantly associated with the outcome, whereas meeting physical activity recommendations remained non-significant. The association between PSQI total score and neck/shoulder symptoms was attenuated and was no longer statistically significant. Overall, the sensitivity analyses showed broadly similar association patterns for smartphone addiction and sedentary time, while the sleep–neck/shoulder symptoms association appeared sensitive to the symptom recall period.

## 4 Discussion

In this study, poor sleep and neck/shoulder symptoms were commonly reported among university students. Higher smartphone addiction and longer sedentary time were consistently associated with poorer sleep quality and more frequent neck/shoulder symptoms, whereas meeting physical activity recommendations was more clearly associated with sleep outcomes than with neck/shoulder symptoms. Although the interaction plots showed descriptively lower predicted outcomes among students who met physical activity recommendations, the formal interaction terms were not statistically significant; therefore, these graphical patterns should be interpreted descriptively and not as evidence of buffering or causal modification.

### 4.1 Smartphone addiction, physical activity, and sleep quality

The prevalence of poor sleep in this study was relatively high and was broadly consistent with the overall burden reported in meta-analyses of sleep problems among Chinese university students and reviews of insomnia symptoms in undergraduate populations worldwide (1, 2). The Smartphone Application-Based Addiction Scale (SABAS) total score was independently and positively associated with both PSQI total score and the prevalence of poor sleep. This finding is consistent with a meta-analysis of mobile phone addiction and sleep disturbance, as well as reviews showing a dose–response relationship between smartphone use duration and self-rated sleep quality (7, 25), and also accords with studies in university students based on objective smartphone-use records (8). Smartphone addiction should be interpreted as one behavioural correlate of poor sleep rather than as the sole explanatory factor. Poor sleep in university students may also be related to academic workload, psychological distress, caffeine intake, sleep habits, nighttime screen exposure, and other lifestyle or environmental factors that were not fully measured in this study. Previous studies have shown that screen use in bed, particularly interactive use, is associated with shorter sleep duration and delayed sleep onset (26), and that nighttime device exposure is linked to poorer sleep outcomes (27).

By contrast, insufficient physical activity and sedentary time did not relate to health outcomes in the same way. Meeting physical activity recommendations was associated with lower PSQI total scores and a lower prevalence of poor sleep, consistent with the broader evidence that higher physical activity is associated with better sleep quality in university populations (28). This finding is also consistent with previous evidence suggesting interrelationships among physical activity, stress, smartphone addiction, and sleep quality (10). Sedentary time, however, showed a more stable adverse association with both sleep and neck/shoulder symptoms. Previous meta-analyses have shown that prolonged sedentary time is associated with a higher risk of insomnia and sleep disturbance (29), and recent systematic reviews have likewise shown that sedentary behaviour is associated with an increased risk of neck pain, with risk rising as exposure duration increases (13, 14). These findings suggest that, in this sample, meeting physical activity recommendations was more consistently associated with sleep-related outcomes, whereas sedentary time was associated with both poor sleep and musculoskeletal discomfort.

### 4.2 Behaviour-sleep correlates of neck/shoulder symptoms

After further adjustment for PSQI total score, smartphone addiction, sedentary time, and sleep quality remained independently associated with neck/shoulder symptoms during the previous 7 days. These findings suggest that neck/shoulder symptoms co-occurred with a broader lifestyle-related pattern involving smartphone addiction, sedentary time, and sleep quality, rather than reflecting isolated postural exposure alone. Previous studies have identified insufficient exercise, prolonged use of electronic devices, sustained head-down posture, staying up late, and a history of neck/shoulder injury as major correlates of neck pain in university students (5). Primary and longitudinal studies have likewise suggested that smartphone addiction, poor sleep quality, and insufficient physical activity are all associated with neck/shoulder discomfort (15, 30, 31). Accordingly, neck/shoulder symptoms should be interpreted as being associated with broader campus lifestyle factors rather than attributed solely to head-down posture.

Meeting physical activity recommendations was not independently associated with neck/shoulder symptoms in the multivariable models (PR = 0.970), and this pattern remained after adjustment for PSQI. In addition, the SABAS total score × physical activity interaction term was not statistically significant. Therefore, this study does not provide statistical evidence that physical activity independently protected against neck/shoulder symptoms or buffered the association between smartphone addiction and neck/shoulder symptoms. Physical activity was more consistently associated with sleep outcomes than with musculoskeletal symptoms. Any apparent separation in the interaction plots at higher SABAS levels should therefore be viewed as descriptive and hypothesis-generating, particularly because fewer observations may be available at extreme SABAS values. Previous studies in Chinese university students have reported interrelationships among physical activity, stress, smartphone addiction, and sleep quality (9, 10). In the present study, however, the formal interaction terms were not statistically significant, so the graphical differences in Figure 3 should be interpreted only as descriptive patterns. These findings suggest that the relationship among smartphone addiction, physical activity, sleep, and neck/shoulder symptoms requires further examination in longitudinal or intervention studies. This pattern is broadly consistent with previous studies in Chinese university students: exercise may moderate the association between smartphone addiction and sleep quality (9), and evening exercise has also been associated with less problematic bedtime smartphone use and lower levels of smartphone addiction (32). Randomized controlled trials and meta-analyses likewise suggest that regular exercise can reduce smartphone addiction (33, 34). Given the high prevalence of sedentary exposure among university students (35) and the continuing increase in the burden of neck pain (4), future campus health promotion research may consider coordinated strategies involving bedtime screen management, interrupting sedentary time between classes, promoting physical activity, and delivering sleep hygiene education, rather than focusing solely on smartphone use or posture.

### 4.3 Distribution of symptom sites and implications for campus health promotion

The findings showed that symptoms reported during both the previous 7 days and the previous 12 months were concentrated mainly in the neck, shoulder, and upper back, providing a clear empirical basis for selecting the neck/shoulder region as the primary outcome in this study. This pattern is consistent with the lifestyle profile commonly seen in university students, in which prolonged screen-based study, sustained sitting, and forward head posture frequently coexist, and it accords with previous evidence. A meta-analysis of risk factors for neck pain in university students identified insufficient exercise, poor sitting posture, prolonged use of electronic devices, sustained head-down posture, staying up late, and a history of neck/shoulder injury as relatively stable correlates (5). More recent reviews further indicate a dose–response relationship between sedentary behaviour and neck pain, with the strongest associations observed for smartphone-related sedentary exposure (14). Excessive smartphone use has also been linked to a higher risk of neck pain, as well as upper-extremity pain and functional limitation (11, 12). Taken together, the clustered distribution across the neck, shoulders, and upper back is consistent with upper-body discomfort co-occurring with screen-based study and sedentary routines, but does not establish a behavioural cause.

These findings suggest that future campus health promotion research may consider sleep hygiene, bedtime screen management, physical activity promotion, sedentary-time interruption, and basic ergonomic guidance together, rather than focusing only on smartphone use or posture correction.

### 4.4 Strengths and limitations

A key strength of this study is the use of established instruments, including the Chinese version of the Smartphone Application-Based Addiction Scale (SABAS-CV), the Global Physical Activity Questionnaire (GPAQ), the Pittsburgh Sleep Quality Index (PSQI), and the Nordic Musculoskeletal Questionnaire (NMQ), to examine sleep quality and neck/shoulder symptoms simultaneously within the same sample. By combining multivariable models with interaction analyses, the study also characterised the joint pattern of smartphone addiction and physical activity, allowing the findings to more closely reflect the real-world structure of health problems in university students.

Several limitations should also be acknowledged. First, the cross-sectional design precludes determination of temporal sequence and causal inference. The study was also not specifically powered to detect small interaction effects, especially at extreme levels of smartphone addiction; therefore, the interaction analyses should be interpreted as exploratory. Therefore, the observed associations should not be interpreted as evidence that smartphone addiction or sedentary behaviour caused poor sleep or neck/shoulder symptoms, or that physical activity reduced these outcomes. Reverse causality is also possible, as students with poorer sleep quality or neck/shoulder symptoms may have been less physically active, spent more time sedentary, or used smartphones more frequently. In addition, although established instruments were used, the PSQI, GPAQ, and NMQ were not revalidated specifically in the present sample, which may affect measurement precision. All main variables were self-reported, which may have introduced recall bias, social desirability bias, and exposure or outcome misclassification. The absence of objective measures, such as smartphone-use logs, accelerometry-based physical activity or sedentary time, actigraphy-based sleep assessment, or ergonomic/postural assessment, may have attenuated or exaggerated the observed associations. Second, the single-university convenience cluster sample limits representativeness and external validity. Students in this institution may differ from the broader population of Chinese university students in regional context, university type, academic workload, campus routines, physical activity opportunities, and screen-based learning requirements. Therefore, the prevalence estimates should not be interpreted as national estimates, and the adjusted PRs should be viewed as associations within this analytic sample rather than directly generalisable estimates for all Chinese university students. Residual confounding is also possible because several relevant factors, including mental health, academic workload, caffeine intake, socioeconomic status, ergonomic conditions, and nighttime screen use, were not measured or included. In addition, all main variables were self-reported and collected using the same questionnaire, which may have introduced recall bias, social desirability bias, exposure or outcome misclassification, and common method variance. Future studies should use multicentre samples and incorporate objective assessments of smartphone use, sleep, physical activity, sedentary behaviour, and anthropometric indicators. In addition, the primary NMQ outcome captured symptoms during the previous 7 days and may not fully reflect recurrent or chronic neck/shoulder symptoms; therefore, 12-month neck/shoulder symptoms were examined in sensitivity analyses. Overall, smartphone addiction, physical activity, sedentary behaviour, sleep quality, and neck/shoulder symptoms are best considered together within a unified campus health promotion framework.

## 5 Conclusion

In this single-university sample, both poor sleep and neck/shoulder symptoms during the previous 7 days were common. Smartphone addiction and sedentary behaviour were consistently associated with poorer sleep quality and more frequent neck/shoulder symptoms. Meeting physical activity recommendations was more consistently associated with sleep outcomes than with neck/shoulder symptoms. Although the interaction plots showed descriptively lower predicted PSQI scores and lower predicted probabilities of neck/shoulder symptoms among students who met physical activity recommendations, the formal interaction terms were not statistically significant. Therefore, these graphical patterns should be interpreted descriptively rather than as evidence of statistically supported effect modification. These association patterns support considering smartphone use, sedentary behaviour, physical activity, and sleep management together in future campus health promotion research.

## Data Availability

The raw data supporting the conclusions of this article will be made available by the authors, without undue reservation.

## Supplementary material

SUPPLEMENTARY FIGURE S1 Conceptual framework linking smartphone addiction, sedentary behaviour, physical activity, sleep quality, and neck/shoulder symptomss. Smartphone addiction, sedentary behaviour, and physical activity were considered as behavioural factors, while sleep quality and neck/shoulder symptomss were considered as health outcomes. Solid lines indicate prespecified association tests in the multivariable models. The dashed line indicates the tested statistical interaction between SABAS total score and meeting physical activity recommendations. The framework was used to guide variable selection and model specification and does not imply causal or temporal ordering because of the cross-sectional study design. SABAS, Smartphone Application-Based Addiction Scale; PA, physical activity; PSQI, Pittsburgh Sleep Quality Index; BMI, body mass index.

## References

[1] Li L Wang YY Wang SB Zhang L Li L Xu DD . Prevalence of sleep disturbances in Chinese university students: a comprehensive Meta-analysis. J Sleep Res. (2018) 27:e12648. doi: 10.1111/jsr.12648, 29383787

[2] Spyridonidis S Lad D Peters H Ellis J Robinson LJ. Global prevalence of insomnia symptoms in undergraduate university students: a systematic review and Meta-analysis. Sleep Adv. (2025) 6. doi: 10.1093/sleepadvances/zpaf083, 41377134 PMC12687938

[3] Castro O Bennie J Vergeer I Bosselut G Biddle SJH. How sedentary are university students? A systematic review and Meta-analysis. Prev Sci. (2020) 21:332–43. doi: 10.1007/s11121-020-01093-8, 31975312

[4] GBD 2021 Neck Pain Collaborators. Global, regional, and national burden of neck pain, 1990–2020, and projections to 2050: a systematic analysis of the Global Burden of Disease Study 2021. Lancet Rheumatol. (2024) 6:e142–55. doi: 10.1016/s2665-9913(23)00321-1 38383088 PMC10897950

[5] Gao Y Chen Z Chen S Wang S Lin J. Risk factors for neck pain in college students: a systematic review and Meta-analysis. BMC Public Health. (2023) 23:1502. doi: 10.1186/s12889-023-16212-7, 37553622 PMC10408143

[6] Sánchez-Fernández M Borda-Mas M. Problematic smartphone use and specific problematic internet uses among university students and associated predictive factors: a systematic review. Educ Inf Technol (Dordr). (2023) 28:7111–204. doi: 10.1007/s10639-022-11437-2, 36465425 PMC9707285

[7] Chu Y Oh Y Gwon M Hwang S Jeong H Kim HW . Dose-response analysis of smartphone usage and self-reported sleep quality: a systematic review and Meta-analysis of observational studies. J Clin Sleep Med. (2023) 19:621–30. doi: 10.5664/jcsm.10392, 36546366 PMC9978438

[8] Yin J Tang X Liu Z Gong Y Yang H Zhang Y. Associations between both smartphone addiction and objectively measured smartphone use and sleep quality and duration among university students: cross-sectional study. JMIR Ment Health. (2025) 12:e77796. doi: 10.2196/77796, 41289567 PMC12646561

[9] Zhu W Liu J Lou H Mu F Li B. Influence of smartphone addiction on sleep quality of college students: the regulatory effect of physical exercise behavior. PLoS One. (2024) 19:e0307162. doi: 10.1371/journal.pone.0307162, 39058670 PMC11280214

[10] Wang J Liu X Xu X Wang H Yang G. The effect of physical activity on sleep quality among Chinese college students: the chain mediating role of stress and smartphone addiction during the Covid-19 pandemic. Psychol Res Behav Manag. (2024) 17:2135–47. doi: 10.2147/PRBM.S462794, 38826679 PMC11143986

[11] Chen YJ Hu CY Wu WT Lee RP Peng CH Yao TK . Association of Smartphone Overuse and Neck Pain: a systematic review and Meta-analysis. Postgrad Med J. (2025) 101:620–6. doi: 10.1093/postmj/qgae200, 39764644

[12] de Jesus Correia F Soares JB Dos Anjos Matos R Pithon KR Ferreira LN de Assunção PL. Smartphone addiction, musculoskeletal pain and functionality in university students - a observational study. Psychol Health Med. (2024) 29:286–96. doi: 10.1080/13548506.2023.2176893, 36803275

[13] Mazaheri-Tehrani S Arefian M Abhari AP Riahi R Vahdatpour B Baradaran Mahdavi S . Sedentary behavior and neck pain in adults: a systematic review and Meta-analysis. Prev Med. (2023) 175:107711. doi: 10.1016/j.ypmed.2023.107711, 37775083

[14] Meng Y Xue Y Yang S Wu F Dong Y. The associations between sedentary behavior and neck pain: a systematic review and Meta-analysis. BMC Public Health. (2025) 25:453. doi: 10.1186/s12889-025-21685-9, 39905389 PMC11796249

[15] Correia IMT Ferreira AS Gomes JFM Reis FJJ Nogueira LAC Meziat-Filho N. Cervical flexion posture during smartphone use was not a risk factor for neck pain, but low sleep quality and insufficient levels of physical activity were. A longitudinal investigation. Braz J Phys Ther. (2025) 29:101258. doi: 10.1016/j.bjpt.2025.101258, 40845624 PMC12398242

[16] von Elm E Altman DG Egger M Pocock SJ Gøtzsche PC Vandenbroucke JP . The strengthening the reporting of observational studies in epidemiology (STROBE) statement: guidelines for reporting observational studies. PLoS Med. (2007) 4:e296. doi: 10.1371/journal.pmed.0040296, 17941714 PMC2020495

[17] Csibi S Griffiths MD Cook B Demetrovics Z Szabo A. The psychometric properties of the smartphone application-based addiction scale (Sabas). Int J Ment Health Addict. (2018) 16:393–403. doi: 10.1007/s11469-017-9787-2, 29670500 PMC5897481

[18] Keating XD Zhou K Liu X Hodges M Liu J Guan J . Reliability and concurrent validity of global physical activity questionnaire (Gpaq): a systematic review. Int J Environ Res Public Health. (2019) 16:4128. doi: 10.3390/ijerph16214128, 31717742 PMC6862218

[19] Bull FC Maslin TS Armstrong T. Global physical activity questionnaire (Gpaq): nine country reliability and validity study. J Phys Act Health. (2009) 6:790–804. doi: 10.1123/jpah.6.6.790, 20101923

[20] Liu X Tang M Hu L Wang A Wu H Zhao G . Reliability and validity of the Pittsburgh sleep quality index. Chin J Psychiatry. (1996) 2:103–7.

[21] Zheng B Li M Wang K Lv J. Analysis of the reliability and validity of the Chinese version of Pittsburgh Sleep Quality Index among medical college students. Beijing Da Xue Xue Bao Yi Xue Ban. (2016) 48:424–8. doi: 10.3969/j.issn.1671-167X.2016.03.009 27318902

[22] Kuorinka I Jonsson B Kilbom A Vinterberg H Biering-Sørensen F Andersson G . Standardised Nordic questionnaires for the analysis of musculoskeletal symptoms. Appl Ergon. (1987) 18:233–7. doi: 10.1016/0003-6870(87)90010-x, 15676628

[23] Fang YX Li SY Zhang YN Zhang P Wu H Wang DH. Test-retest reliability of Nordic musculoskeletal questionnaire in nurses. Zhonghua Lao Dong Wei Sheng Zhi Ye Bing Za Zhi. (2013) 31:753–8. doi: 10.3760/cma.j.issn.1001-9391.2013.10.008 24148953

[24] Zou G. A modified Poisson regression approach to prospective studies with binary data. Am J Epidemiol. (2004) 159:702–6. doi: 10.1093/aje/kwh090, 15033648

[25] Zhang J Zhang X Zhang K Lu X Yuan G Yang H . An updated of Meta-analysis on the relationship between Mobile phone addiction and sleep disorder. J Affect Disord. (2022) 305:94–101. doi: 10.1016/j.jad.2022.02.008, 35149136

[26] Brosnan B Haszard JJ Meredith-Jones KA Wickham SR Galland BC Taylor RW. Screen use at bedtime and sleep duration and quality among youths. JAMA Pediatr. (2024) 178:1147–54. doi: 10.1001/jamapediatrics.2024.2914, 39226046 PMC11372655

[27] Carter B Rees P Hale L Bhattacharjee D Paradkar MS. Association between portable screen-based media device access or use and sleep outcomes: a systematic review and Meta-analysis. JAMA Pediatr. (2016) 170:1202–8. doi: 10.1001/jamapediatrics.2016.2341, 27802500 PMC5380441

[28] Memon AR Gupta CC Crowther ME Ferguson SA Tuckwell GA Vincent GE. Sleep and physical activity in university students: a systematic review and Meta-analysis. Sleep Med Rev. (2021) 58:101482. doi: 10.1016/j.smrv.2021.101482, 33864990

[29] Yang Y Shin JC Li D An R. Sedentary behavior and sleep problems: a systematic review and Meta-analysis. Int J Behav Med. (2017) 24:481–92. doi: 10.1007/s12529-016-9609-0, 27830446

[30] Alghadir AH Gabr SA Rizk AA Alghadir T Alghadir F Iqbal A. Smartphone addiction and musculoskeletal associated disorders in university students: biomechanical measures and questionnaire survey analysis. Eur J Med Res. (2025) 30:274. doi: 10.1186/s40001-025-02413-w, 40229835 PMC11998459

[31] Intaruk R Sengsoon P. Differences in posture, neck angle, and body discomfort during various electronic device usage with virtual classroom. Int J Environ Res Public Health. (2025) 22:1418. doi: 10.3390/ijerph22091418, 41007562 PMC12469765

[32] Su Y Li H Jiang S Li Y Li Y Zhang G. The relationship between nighttime exercise and problematic smartphone use before sleep and associated health issues: a cross-sectional study. BMC Public Health. (2024) 24:590. doi: 10.1186/s12889-024-18100-0, 38395834 PMC10893754

[33] Li Z Xia X Sun Q Li Y. Exercise intervention to reduce Mobile phone addiction in adolescents: a systematic review and Meta-analysis of randomized controlled trials. Front Psychol. (2023) 14:1294116. doi: 10.3389/fpsyg.2023.1294116, 38192396 PMC10773895

[34] Xiao T Jiao C Yao J Yang L Zhang Y Liu S . Effects of basketball and Baduanjin exercise interventions on problematic smartphone use and mental health among college students: a randomized controlled trial. Evid Based Complement Alternat Med. (2021) 2021:1. doi: 10.1155/2021/8880716, 33574886 PMC7864751

[35] Lynch J O'Donoghue G Peiris CL. Classroom movement breaks and physically active learning are feasible, reduce sedentary behaviour and fatigue, and may increase focus in university students: a systematic review and meta-analysis. Int J Environ Res Public Health. (2022) 19:7775. doi: 10.3390/ijerph19137775 35805432 PMC9265656

[36] Guo S Sun W Liu C Wu S. Structural validity of the Pittsburgh sleep quality index in Chinese undergraduate students. Front Psychol. (2016) 7:1126. doi: 10.3389/fpsyg.2016.01126, 27551270 PMC4976124

[37] Castro O Bennie J Vergeer I Bosselut G Biddle SJH. Correlates of sedentary behaviour in university students: a systematic review. Prev Med. (2018) 116:194–202. doi: 10.1016/j.ypmed.2018.09.016, 30266213

