# Supplementary material for: Associations of smartphone addiction and physical activity with sleep quality and neck/shoulder symptoms in university students: a cross-sectional study
Source: Front Public Health. 2026 Jun 22;14:1848640. doi: 10.3389/fpubh.2026.1848640 (PMC13333704; doi:10.3389/fpubh.2026.1848640)
Supplement: Supplementary file 2 [file Table_1.docx]

# Supplementary Table S1. Sensitivity analyses using alternative outcome definitions

## Panel A. Poor sleep redefined as PSQI > 5

## Panel B. Neck/shoulder symptoms redefined as symptoms during the previous 12 months

| **Panel** | **Variable** | **PR (95% CI)** | **P value** |
| --- | --- | --- | --- |
| Panel A: Poor sleep (PSQI > 5) | sabas_total | 1.029 (1.019, 1.039) | <0.001 |
|  | meet_pa_guideline1 | 0.864 (0.781, 0.955) | 0.004 |
|  | sedentary_hours_day | 1.066 (1.030, 1.103) | <0.001 |
|  | sex2 | 1.158 (1.034, 1.297) | 0.011 |
|  | age | 0.891 (0.816, 0.972) | 0.009 |
|  | grade2 | 1.084 (0.902, 1.302) | 0.390 |
|  | grade3 | 1.249 (1.004, 1.554) | 0.046 |
|  | grade4 | 1.163 (0.864, 1.566) | 0.320 |
|  | grade5 | 1.629 (1.046, 2.538) | 0.031 |
|  | bmi | 1.011 (0.995, 1.027) | 0.175 |
|  | smoking_30d1 | 0.953 (0.777, 1.169) | 0.647 |
|  | drinking_30d1 | 0.892 (0.747, 1.064) | 0.204 |
|  | chronic_disease1 | 1.022 (0.826, 1.264) | 0.843 |
|  | neck_injury_history1 | 1.239 (1.000, 1.535) | 0.050 |
| Panel B: Neck/shoulder symptoms (12 months) | sabas_total | 1.026 (1.013, 1.039) | <0.001 |
|  | meet_pa_guideline1 | 0.964 (0.843, 1.102) | 0.589 |
|  | sedentary_hours_day | 1.071 (1.028, 1.115) | 0.001 |
|  | psqi_total | 1.022 (0.997, 1.047) | 0.090 |
|  | sex2 | 0.954 (0.837, 1.087) | 0.479 |
|  | age | 0.947 (0.853, 1.051) | 0.306 |
|  | grade2 | 0.946 (0.761, 1.176) | 0.617 |
|  | grade3 | 1.070 (0.828, 1.382) | 0.606 |
|  | grade4 | 1.153 (0.814, 1.633) | 0.423 |
|  | grade5 | 1.126 (0.660, 1.923) | 0.663 |
|  | bmi | 1.006 (0.986, 1.025) | 0.567 |
|  | smoking_30d1 | 0.939 (0.720, 1.225) | 0.642 |
|  | drinking_30d1 | 1.040 (0.859, 1.260) | 0.687 |
|  | chronic_disease1 | 0.991 (0.755, 1.302) | 0.950 |
|  | neck_injury_history1 | 1.213 (0.977, 1.505) | 0.080 |

Note: PR, prevalence ratio; CI, confidence interval; PSQI, Pittsburgh Sleep Quality Index. Panel A shows the robust Poisson regression model after redefining poor sleep as PSQI > 5. Panel B shows the robust Poisson regression model after redefining neck/shoulder symptoms as symptoms reported during the previous 12 months. The same covariate adjustment schemes as in the primary analyses were used.
