# Supplementary material for: Associations of smartphone addiction and physical activity with sleep quality and neck/shoulder symptoms in university students: a cross-sectional study
Source: Front Public Health. 2026 Jun 22;14:1848640. doi: 10.3389/fpubh.2026.1848640 (PMC13333704; doi:10.3389/fpubh.2026.1848640)
Supplement: Supplementary file 3 [file Table_2.docx]

# Supplementary Table S2. Interaction analyses between smartphone addiction and meeting physical activity recommendations

| **Outcome** | **Model** | **Interaction term** | **Estimate** | **95% CI** | **P value** | **Interpretation** |
| --- | --- | --- | --- | --- | --- | --- |
| Pittsburgh Sleep Quality Index total score | Linear regression | SABAS total score × meeting physical activity recommendations | β = -0.004 | -0.004; 95% CI -0.068 to 0.060 | 0.895 | The interaction term was not statistically significant; therefore, graphical differences should be interpreted descriptively rather than as evidence of statistically supported effect modification. |
| Neck/shoulder symptoms during the previous 7 days | Robust Poisson regression | SABAS total score × meeting physical activity recommendations | PR = 1.005 | PR = 1.007; 95% CI 0.984 to 1.031; | 0.536 | The interaction term was not statistically significant; therefore, graphical differences should be interpreted descriptively rather than as evidence of statistically supported effect modification. |

Note: β was reported for the linear regression model with Pittsburgh Sleep Quality Index total score as the outcome. PR for interaction was reported for the robust Poisson regression model with neck/shoulder symptoms during the previous 7 days as the outcome. Models included the main effects of SABAS total score and meeting physical activity recommendations and were adjusted for sex, age, academic year, body mass index, smoking during the previous 30 days, alcohol consumption during the previous 30 days, chronic disease, history of neck/shoulder injury, and sedentary time. The neck/shoulder symptom model was additionally adjusted for PSQI total score. SABAS, Smartphone Application-Based Addiction Scale; PSQI, Pittsburgh Sleep Quality Index; PR, prevalence ratio; CI, confidence interval.
