# Supplementary material for: Associations of smartphone addiction and physical activity with sleep quality and neck/shoulder symptoms in university students: a cross-sectional study
Source: Front Public Health. 2026 Jun 22;14:1848640. doi: 10.3389/fpubh.2026.1848640 (PMC13333704; doi:10.3389/fpubh.2026.1848640)
Supplement: Supplementary file 4 [file Table_3.docx]

# Supplementary Table S3. Sensitivity analyses using alternative physical activity specifications

## Panel A. Physical activity volume as continuous variable, per 1000 MET-min/week

## Panel B. Physical activity volume as quartiles

## Panel C. Exploratory quadratic term for physical activity volume

| **Panel** | **Outcome** | **Model** | **Physical activity specification** | **Estimate** | **95% CI** | **P value** |
| --- | --- | --- | --- | --- | --- | --- |
| A | Pittsburgh Sleep Quality Index total score | Linear regression | Per 1000 MET-min/week | β = -0.261 | -0.393 to -0.129 | <0.001 |
|  | Poor sleep, PSQI > 7 | Robust Poisson regression | Per 1000 MET-min/week | PR = 0.909 | 0.846 to 0.977 | 0.010 |
|  | Neck/shoulder symptoms during the previous 7 days | Robust Poisson regression | Per 1000 MET-min/week | PR = 0.960 | 0.900 to 1.024 | 0.215 |
| B | Pittsburgh Sleep Quality Index total score | Linear regression | Q2 vs Q1 | β = -1.065 | -1.661 to -0.469 | <0.001 |
|  | Pittsburgh Sleep Quality Index total score | Linear regression | Q3 vs Q1 | β = -1.094 | -1.706 to -0.481 | <0.001 |
|  | Pittsburgh Sleep Quality Index total score | Linear regression | Q4 vs Q1 | β = -1.529 | -2.146 to -0.912 | <0.001 |
|  | Poor sleep, PSQI > 7 | Robust Poisson regression | Q2 vs Q1 | PR = 0.835 | 0.678 to 1.027 | 0.088 |
|  | Poor sleep, PSQI > 7 | Robust Poisson regression | Q3 vs Q1 | PR = 0.764 | 0.586 to 0.995 | 0.046 |
|  | Poor sleep, PSQI > 7 | Robust Poisson regression | Q4 vs Q1 | PR = 0.704 | 0.541 to 0.917 | 0.009 |
|  | Neck/shoulder symptoms during the previous 7 days | Robust Poisson regression | Q2 vs Q1 | PR = 1.016 | 0.817 to 1.263 | 0.889 |
|  | Neck/shoulder symptoms during the previous 7 days | Robust Poisson regression | Q3 vs Q1 | PR = 1.062 | 0.836 to 1.351 | 0.621 |
|  | Neck/shoulder symptoms during the previous 7 days | Robust Poisson regression | Q4 vs Q1 | PR = 0.919 | 0.702 to 1.202 | 0.537 |
| C | Pittsburgh Sleep Quality Index total score | Linear regression | Quadratic term (per 1000 MET-min/week)² | β = 0.065 | 0.015 to 0.115 | 0.010 |
|  | Poor sleep, PSQI > 7 | Robust Poisson regression | Quadratic term (per 1000 MET-min/week)² | PR = 0.999 | 0.975 to 1.023 | 0.912 |
|  | Neck/shoulder symptoms during the previous 7 days | Robust Poisson regression | Quadratic term (per 1000 MET-min/week)² | PR = 0.979 | 0.945 to 1.015 | 0.248 |

Note: Panel A reports models in which total physical activity volume was analysed as a continuous variable per 1000 MET-min/week. Panel B reports models in which total physical activity volume was analysed by quartiles, with Q1 as the reference group. Panel C reports exploratory models including a quadratic term for total physical activity volume. β was reported for linear regression models, and PR was reported for robust Poisson regression models. All models were adjusted for SABAS total score, sedentary time, sex, age, academic year, body mass index, smoking during the previous 30 days, alcohol consumption during the previous 30 days, chronic disease, and history of neck/shoulder injury. The neck/shoulder symptom models were additionally adjusted for PSQI total score. SABAS, Smartphone Application-Based Addiction Scale; PSQI, Pittsburgh Sleep Quality Index; PR, prevalence ratio; CI, confidence interval.
