# Supplementary material for: Associations of smartphone addiction and physical activity with sleep quality and neck/shoulder symptoms in university students: a cross-sectional study
Source: Front Public Health. 2026 Jun 22;14:1848640. doi: 10.3389/fpubh.2026.1848640 (PMC13333704; doi:10.3389/fpubh.2026.1848640)
Supplement: Supplementary file 5 [file Table_4.docx]

Supplementary Table S4. FDR-adjusted primary analyses and Poisson model-stability checks

Panel A: FDR-adjusted primary inferential tests

| **Outcome** | **Model** | **Term** | **Estimate** | **95% CI** | **P value** | **FDR.adjusted.q.value** | **Interpretation** |
| --- | --- | --- | --- | --- | --- | --- | --- |
| PSQI total | Linear regression | sabas_total | 0.163 | (0.129, 0.198) | <0.001 | <0.001 | Significant after FDR correction |
| PSQI total | Linear regression | sedentary_hours_day | 0.427 | (0.301, 0.552) | <0.001 | <0.001 | Significant after FDR correction |
| PSQI total | Linear regression | meet_pa_guideline1 | -1.344 | (-1.804, -0.883) | <0.001 | <0.001 | Significant after FDR correction |
| Poor sleep (PSQI > 7) | Poisson regression | sabas_total | 1.055 | (1.039, 1.071) | <0.001 | <0.001 | Significant after FDR correction |
| Poor sleep (PSQI > 7) | Poisson regression | sedentary_hours_day | 1.130 | (1.074, 1.189) | <0.001 | <0.001 | Significant after FDR correction |
| Poor sleep (PSQI > 7) | Poisson regression | meet_pa_guideline1 | 0.742 | (0.628, 0.877) | <0.001 | <0.001 | Significant after FDR correction |
| Neck/shoulder symptoms (past 7 days) | Poisson regression | sabas_total | 1.019 | (1.003, 1.034) | 0.019 | 0.025 | Significant after FDR correction |
| Neck/shoulder symptoms (past 7 days) | Poisson regression | sedentary_hours_day | 1.103 | (1.047, 1.161) | <0.001 | <0.001 | Significant after FDR correction |
| Neck/shoulder symptoms (past 7 days) | Poisson regression | psqi_total | 1.049 | (1.016, 1.083) | 0.003 | 0.005 | Significant after FDR correction |
| Neck/shoulder symptoms (past 7 days) | Poisson regression | meet_pa_guideline1 | 0.970 | (0.811, 1.159) | 0.736 | 0.803 | Not significant after FDR correction |
| PSQI total | Linear regression (interaction) | sabas_total:meet_pa_guideline1 | -0.004 | (-0.068, 0.060) | 0.895 | 0.895 | Not significant after FDR correction |
| Neck/shoulder symptoms (past 7 days) | Poisson regression (interaction) | sabas_total:meet_pa_guideline1 | 1.007 | (0.984, 1.031) | 0.536 | 0.643 | Not significant after FDR correction |

Panel B: Poisson model-stability checks

| **Model** | **Outcome_events** | **Non_events** | **Estimated_coefficients_excluding_intercept** | **Events_per_coefficient** | **Non_events_per_coefficient** | **Model_converged** | **Sparse_cell_check** | **Sensitivity_analysis** |
| --- | --- | --- | --- | --- | --- | --- | --- | --- |
| Model 2: Poor sleep (PSQI > 7) | 256 | 329 | 16 | 16.00 | 20.56 | Yes | No major sparse-cell concern | Materially similar estimates after collapsing smoking and alcohol use |
| Model 3: Neck/shoulder symptoms (past 7 days) | 270 | 315 | 17 | 15.88 | 18.53 | Yes | No major sparse-cell concern | Materially similar estimates after collapsing smoking and alcohol use |
